# Supplementary material for: Geometrical model of lobular structure and its importance for the liver perfusion analysis
Source: PLoS One. 2021 Dec 2;16(12):e0260068. doi: 10.1371/journal.pone.0260068 (PMC8638901; doi:10.1371/journal.pone.0260068)
Supplement: Attachment — Submitted filename: Reply-to-Referee-PLOSone2021-R.pdf [file pone.0260068.s001.pdf]

Reply to the Reviewer comments

Title of the manuscript: **Geometrical model of lobular structure and its importance for the liver perfusion analysis – Revised version R1**
Authors: Eduard Rohan, Jana Turjanicová and Václav Liška

We thank the reviewer for his/her comments and suggestions to clarify some issues of the paper. Below we provide our reactions to the particular Reviewer's comments and specify modifications carried out in the paper.

Reviewer: This manuscript is concerned with the modelling of blood circulation in the liver tissue. The liver has fundamental units (the lobules), the structure of which has been considered in various models of liver blood perfusion. In the present manuscript the authors propose the use of a smaller unit, which they call representative periodic cell (LRPC). They discuss how this enables to use homogenization methods to derive macroscopic liver perfusion properties.

Reply: Indeed, the main purpose of the paper is to present a geometric framework for building locally periodic representative cells (volumes) which enable to apply more or less rigorous homogenization (upscaling) techniques providing convenient "macroscopic" models of the perfused liver parenchyma. By no means the paper claims to describe a "complex model of perfusion" which captures the hierarchical arrangement of the liver perfusion system. However, the paper provides an important part of such a model which, up to our knowledge, has not been described so far at a sufficient level of rigor an potential compatibility with the other parts. The suggested LRPC combined with a homogenization based flow model, such as the one called the Double-permeability Darcy flow model, is relevant to a mesoscopic scale. On one hand, it incorporates a model describing microcirculation in the sinusoidal porosity (micro-scale). On the other hand, it must be connected to a model describing the upper hierarchies of the perfusion system associated with branching structures – perfusion trees – associated with PV, HV and HA.

Reviewer: The paper is quite well written but, in my view, many parts are unnecessarily over complicated. This makes it reading the manuscript more difficult that it ought to be and, ultimately, makes it hard to grasp some of the key features of the work. I would recommend that the authors simplify the exposition, which does not necessarily imply that the presentation needs to be less formal and rigorous.

Reply: The only "technical part" containing mathematical expressions constitutes the major part and the principal contribution of the paper. It really happens that some geometrical relationships require a certain formalism to be adhered, otherwise ambiguities emerge and by the consequence complicate understanding the result. Nevertheless, significant modifications in the presentation of this part has been made; in particular, some of the definitions of the basic geometrical entities has been moved to the Appendix and many parts of the text have been reorganized. Some more explanations have been added, including a new figure to illustrate how the LRPC is obtained using translations in the primary and dual lattices which are associated with the central (hepatic vein) and portal vein precapillary vessels.

Reviewer: Having said this, I have some fundamental doubts about the work that I explain in the following.

- To my knowledge most of the resistance to the flow occurs at the level of the microcirculation, which means in the sinusoids. The authors' approach allows one to compute the permeability of small portal and hepatic veins, but obviously not of the sinusoids. If it is true that flow resistance is concentrated in the sinusoids, this spoils the usefulness of determining the permeability of larger vessels.

Reply: We understand this point and agree that the flow in sinusoids deserves a special attention, since the capillary network (the sinusoids) presents the major flow resistance. Nonetheless, this does not contradicts the paper contribution and the importance of LRPC in this context. A

sinusoidal model is a complementary part to be supplied. It should be emphasized, that the flow in capillary network is cumbersome problem because of the fluid heterogeneity. Within the proposed concept, the sinusoidal porosity is represented by a permeability which is a tensorial Y-periodic, but inhomogeneous function defined in subdomain Y_m. The paper is modified in the respective part to clarify this point.

However, we do not share the implication involved in the Reviewer's statement *"If it is true that flow resistance is concentrated in the sinusoids, this spoils the usefulness of determining the permeability of larger vessels"*. If the upper hierarchy including the precapillary (larger vessels) is equally important for the model correctness. Moreover, the networks (namely the PV and HV ones) of larger vessels provide an important part of the hierarchical perfusion system which cannot be skipped or oversimplified. In nonpathological situations, it ensures evenly distributed supply of blood to the capillary system. The desired model should provide natural means for describing pathological dysfunctions and morphological irregularities though anatomically and physiologically consistent. We believe that our concept provide a consistent framework to meet such requirements.

This issue has be reflected in the revised manuscript to a large extent, comments have been included at many parts and namely in the Discussion.

Reviewer: - I also ave some doubts about the fact that the authors' approach allows one to model macroscopic variations of the perfusion of liver tissue. Blood is delivered to microscopic structures through large vessels, within which the resistance is relatively small. Thus the view of a macroscopic percolation through a porous medium constituted by a large number of LRPCs is not very realistic, in my opinion. Blood is transported to "groups" of LRPCs through large vessels and only locally at the microscale blood flow can be thought of as the flow in a porous medium. In order to understand possible perfusion depletion of certain tissue regions one has to study blood flow in relatively large vessels.

Reply: As already indicated in our reply above, the LRPC provides just one, though a very important level of the complex model. We describe the geometrical (topological) model of the parenchyma which needs to be connected to A) a mesoscopic flow model which can be upscaled (homogenized) to obtain its macroscopic continuum representation (Note that this includes the above discussed model of the sinusoidal porosity); B) a macroscopic flow model accounting for the hierarchical blood flow which provides (desirably) evenly distributed supply and drainage of the blood into and from the lobular structure (the parenchyma blood perfusion). The latter can be realized by a reduced 1D modelling (Poiseuille and/or Bernoulli) of the 3D branching vessel structures, alternatively combined with a multicompartment model. Such a model has been described in our paper [17] (new ordering in the list), where the "lobular level" was treated by a simple phenomenological model which can be replaced by the one based on the LRPC and the homogenization.

We have rewritten a part of the text in Introduction and Conclusion to clarify this concept and the issues for complementary works.

Reviewer: I think the authors should carefully consider the above points before the manuscript can be considered for publication on PLOS ONE.

Reply: We believe that the Reviewer's comments were respected in the revised paper and helped to clarify the meaning of the LRPC in the context of the complex perfusion model and related extensions, such as those aimed at describing the tissue evolution due the oxygen, nutrients and waste transport.

Survey of modifications made in the revised manuscript All significant modifications made through the whole text are displayed in blue color.

- Many paragraphs in the text have been rewritten to clarify the main contribution of the paper and to better explain the role of the LRPC in the context of the "whole liver perfusion model".
- New references were cited, [15,18,29] (new numbering).

- Some nomenclature and definitions of the geometrical entities are postponed in the Appendix.
- Figures have been reorganized, new figure (now Fig. 6) is now included.
- The Discussion has been rewritten significantly to respect the issues pointed out by the Referee.
